# Beyond Tumor Diameter: Exploratory Cohort-Derived Calcitonin Secretory Categories and Invasive Pathology in Medullary Thyroid Carcinoma

**DOI:** 10.3390/curroncol33090533

**Published:** 2026-09-02

**Authors:** Adem Ozcan, Gizem Gunes, Ali Bal, Abdulkadir Unsal, Furkan Savas, Mustafa Omer Yazicioglu

**Affiliations:** 1 Department of Surgical Oncology, Ankara Bilkent City Hospital, Ankara 06800, Türkiye; 2 Department of General Surgery, Ankara Bilkent City Hospital, Ankara 06800, Türkiye

**Keywords:** medullary thyroid carcinoma, calcitonin, tumor diameter, biochemical heterogeneity, lymphovascular invasion, perineural invasion

## Abstract

**Background:** Serum calcitonin is commonly interpreted as a marker of tumor burden in medullary thyroid carcinoma (MTC), but patients with comparable tumor diameters may show markedly different calcitonin levels. This exploratory study aimed to derive cohort-specific, tumor diameter–adjusted calcitonin secretory categories and examine their relationship with invasive pathological features. **Methods:** This retrospective single-center study included 70 unique patients with histopathologically confirmed MTC. Seventeen patients with preoperative serum calcitonin below the assay reporting limit (<2 pg/mL) were evaluated separately. In 53 patients with detectable calcitonin, residuals from a log-linear model of calcitonin according to dominant tumor diameter were divided into tertiles to define exploratory hyposecretory, normosecretory, and hypersecretory categories. Sensitivity analyses accounted for sex, metastatic lymph-node count, documented distant metastatic disease, and restriction to patients with pN0 disease and no documented distant metastasis. **Results:** Preoperative calcitonin correlated with dominant tumor diameter (Spearman rho = 0.644, *p* < 0.001), and the primary model explained 45.2% of calcitonin variability (R^2^ = 0.452). The cohort-derived categories included 18 hyposecretory, 17 normosecretory, and 18 hypersecretory tumors. In a hypothesis-driven exploratory contrast, lymphovascular invasion (55.6% vs. 25.7%; nominal *p* = 0.040) and perineural invasion (33.3% vs. 8.6%; nominal *p* = 0.048) were more frequent in the hypersecretory category. However, the global three-group comparisons were not statistically significant (*p* = 0.135 and *p* = 0.117, respectively), and both false-discovery-rate-adjusted q values were 0.072. The tumor diameter–calcitonin relationship remained significant after adjustment for sex and metastatic burden and in the pN0 subgroup without documented distant metastasis. **Conclusions:** The exploratory, cohort-derived hypersecretory category showed hypothesis-generating enrichment for lymphovascular and perineural invasion, but these associations did not meet the false-discovery-rate-adjusted significance threshold. External validation is required before these categories can be considered biologically established or clinically applicable.

## Simple Summary

Medullary thyroid carcinoma (MTC) is a rare thyroid malignancy in which serum calcitonin is generally interpreted as a marker of tumor burden. However, tumors of comparable diameter may produce markedly different calcitonin levels. In this retrospective study of 70 patients, we derived exploratory, cohort-specific secretory categories by modeling preoperative calcitonin relative to dominant tumor diameter. Seventeen patients with serum calcitonin below the laboratory reporting limit were evaluated separately, while 53 patients with detectable calcitonin were categorized as hyposecretory, normosecretory, or hypersecretory. Lymphovascular and perineural invasion were more frequent in the hypersecretory category in an exploratory binary comparison, but the primary associations did not meet the false-discovery-rate-adjusted significance threshold. Sensitivity analyses accounting for sex and documented nodal and distant metastatic burden supported the stability of the tumor diameter–calcitonin relationship. These cohort-derived categories are hypothesis-generating and require external validation before clinical application.

## 1. Introduction

Medullary thyroid carcinoma (MTC) is a distinct neuroendocrine malignancy of thyroid parafollicular C-cell origin, representing a small proportion of thyroid cancers but accounting for a disproportionate burden of thyroid cancer–related morbidity because of its variable biological behavior, early nodal dissemination in selected patients, and potential for persistent biochemical disease [1,2,3,4]. Unlike differentiated thyroid carcinoma, MTC is not iodine-avid and its clinical management relies heavily on integration of biochemical, genetic, surgical, and pathological information [1,2]. Although germline or somatic RET alterations define a major biological axis in hereditary and sporadic disease, the clinical phenotype of MTC remains heterogeneous even among tumors of similar size and stage [1,3,4]. This heterogeneity is particularly evident in the broad spectrum ranging from incidentally detected micro-MTC to tumors with invasive pathological features and distant dissemination.

Serum calcitonin remains the central disease-specific biomarker in MTC. It is used in diagnostic work-up, postoperative surveillance, assessment of biochemical cure, and interpretation of disease burden [2,5,6]. Carcinoembryonic antigen (CEA) provides complementary information and may become particularly relevant when calcitonin kinetics and CEA kinetics diverge, a situation often interpreted as a marker of altered tumor biology or partial dedifferentiation [6,7,8]. Current guidance emphasizes the value of careful calcitonin measurement and interpretation, because analytical variability, assay-specific lower reporting limits, and clinical context can materially influence decision-making [2,5]. Basal serum calcitonin concentrations are also sex-dependent, with higher physiological concentrations generally observed in men. Accordingly, models based on absolute circulating calcitonin may be influenced by sex unless this source of variation is examined explicitly [9]. Therefore, calcitonin should be understood not only as a laboratory value but also as an integrated biochemical expression of tumor burden, C-cell differentiation, and secretory activity.

The conventional interpretation of calcitonin in MTC is largely tumor-burden based: higher calcitonin concentrations are generally associated with larger tumors, greater disease extent, and a higher likelihood of persistent or progressive disease [6,7,10,11,12,13]. Several contemporary studies have reinforced the relationship between preoperative or postoperative calcitonin levels and clinically relevant disease states, including metastatic burden, biochemical persistence, and long-term outcomes [10,11,12,13]. In addition, calcitonin and CEA doubling times have been validated as prognostic indicators in patients with persistent or recurrent disease, supporting the concept that biomarker kinetics capture clinically meaningful tumor behavior [7]. However, these population-level associations do not fully explain the marked interpatient variability observed in daily practice. Patients with comparable primary tumor diameters may present with substantially different calcitonin levels, suggesting that calcitonin secretion is not merely a passive function of tumor diameter.

This discordance is biologically plausible. MTC cells may differ in their degree of neuroendocrine differentiation, secretory granule activity, peptide processing, and immunophenotypic expression of calcitonin and CEA [6,8,14,15]. Rare calcitonin-negative or calcitonin-low MTCs further demonstrate that measurable tumor burden and calcitonin secretion can become dissociated [15]. Such cases challenge the assumption that serum calcitonin is universally proportional to anatomical tumor burden. Conversely, some tumors may secrete disproportionately high amounts of calcitonin relative to their diameter, potentially reflecting a hypersecretory biochemical category. Together, these observations raise an important unresolved question: whether tumor diameter–adjusted calcitonin secretion can delineate reproducible MTC secretory categories.

Pathological features provide an essential framework for evaluating this possibility. Lymphovascular invasion, perineural invasion, capsular invasion, extrathyroidal extension, and tumor necrosis reflect invasive or adverse tumor biology beyond diameter alone. Contemporary pathological frameworks recognize that tumor grade and adverse microscopic features provide information not captured by size-based staging alone [16,17]. The International Medullary Thyroid Carcinoma Grading System defines high-grade MTC by the presence of at least one of the following: a mitotic index of ≥5 per 2 mm^2^, a Ki-67 proliferative index of ≥5%, or tumor necrosis [16,17]. However, whether biochemical secretory behavior, after adjustment for dominant tumor diameter, corresponds to these adverse pathological features remains insufficiently characterized.

A dominant-tumor-diameter–adjusted calcitonin secretion framework may therefore offer a clinically interpretable method for studying biochemical heterogeneity in MTC. Instead of evaluating calcitonin as an isolated absolute value, modeling calcitonin relative to dominant tumor diameter allows identification of tumors that secrete less, approximately as expected, or more calcitonin than predicted for their diameter. This approach separates the available measure of primary anatomical tumor burden from biochemical secretory behavior and may delineate hyposecretory, normosecretory, and hypersecretory categories. Importantly, such a framework does not aim to replace established staging, genetic assessment, or postoperative dynamic risk evaluation [2,4,18,19]. Rather, it provides a complementary analytical perspective for understanding why tumors of similar diameter may show divergent biochemical profiles.

The present study was designed to evaluate this concept in a retrospective cohort of patients with histopathologically confirmed MTC. We aimed to derive exploratory, cohort-specific secretory categories using preoperative calcitonin and dominant tumor diameter and to examine their relationship with invasive pathological features. Because circulating calcitonin may also be influenced by sex and metastatic disease burden, additional sensitivity analyses evaluated sex-adjusted secretion, metastatic lymph-node count, documented distant metastatic disease, and the tumor diameter–calcitonin relationship in patients with pN0 disease and no documented distant metastasis.

## 2. Materials and Methods

### 2.1. Study Design and Ethical Approval

This study was designed as a retrospective, single-center, observational cohort study deriving exploratory, cohort-specific, tumor diameter–adjusted calcitonin secretory categories in patients with medullary thyroid carcinoma (MTC).

The study protocol was reviewed and approved by the Ankara Bilkent City Hospital Scientific and Ethical Evaluation Committee for Medical Research, Ethics Committee No. 1. Ethical approval was granted under approval number TABED 1-26-2669, dated 17 June 2026. The study was conducted in accordance with the principles of the Declaration of Helsinki and relevant national regulations for retrospective clinical research. Because the study was retrospective and used data obtained during routine diagnostic, surgical, biochemical, and pathological care, the requirement for written informed consent was waived by the ethics committee.

The study was reported in accordance with the Strengthening the Reporting of Observational Studies in Epidemiology (STROBE) recommendations for observational cohort studies.

### 2.2. Study Population

The institutional database and electronic medical records were retrospectively reviewed to identify patients who underwent surgical treatment for histopathologically confirmed MTC between 2020 and 2026. Eligible patients were identified from final pathology reports, operative records, laboratory records, and institutional electronic medical files.

Initially, all patient records with a diagnosis of MTC were screened. Patient-level duplication was checked using institutional archive numbers and demographic identifiers. When duplicate entries were detected, only one index patient-level record was retained for analysis. After duplicate patient-level records were removed, the final source cohort consisted of 70 unique patients with histopathologically confirmed MTC.

The primary analytic cohort was defined after assessment of preoperative calcitonin detectability. Seventeen patients had preoperative serum calcitonin values reported by the institutional laboratory as <2 pg/mL, representing the lower laboratory reporting limit available in the historical records. Because these values were left-censored below the reporting limit and could not be used reliably as continuous measurements in log-linear modeling, these patients were excluded from the primary continuous secretory-category analysis and were described separately as an exploratory serum-calcitonin-undetectable subgroup. The primary tumor diameter–adjusted calcitonin category analysis was therefore performed in 53 patients with detectable preoperative calcitonin values.

### 2.3. Inclusion and Exclusion Criteria

Patients were included if they met all of the following criteria:

1. Histopathological diagnosis of medullary thyroid carcinoma;
2. Surgical treatment performed at the study institution;
3. Available preoperative serum calcitonin measurement;
4. Available preoperative serum carcinoembryonic antigen (CEA) measurement;
5. Available pathological tumor diameter;
6. Available pathological assessment of invasive tumor features.

Patients were excluded from the primary continuous secretory category analysis if they had any of the following:

1. Undetectable preoperative calcitonin below the assay reporting limit, reported as <2 pg/mL;
2. Missing pathological tumor diameter;
3. Duplicate patient-level records;
4. Non-medullary thyroid malignancy without a confirmed MTC component;
5. Incomplete data preventing classification of calcitonin secretory category.

Patients with serum calcitonin below the assay reporting limit were not removed from the overall study description. They were analyzed separately as a descriptive subgroup because serum calcitonin below the reporting limit in histologically confirmed MTC may represent a clinically relevant biochemical subgroup, while the censored nature of the laboratory result prevents valid continuous modeling.

### 2.4. Surgical Treatment and Neck Dissection

Sixty-nine patients underwent total thyroidectomy, and one patient underwent right thyroid lobectomy. Central and/or lateral compartment neck dissection was performed in 45 patients according to preoperative imaging, biochemical findings, clinical nodal suspicion, previous operative history, and intraoperative assessment. Neck dissection was not mandatory for inclusion in the study. At least one lymph node was retrieved and examined pathologically in 58 patients.

### 2.5. Data Collection

Data were collected retrospectively from the hospital electronic medical record system, laboratory information system, operative reports, and final pathology reports. Data extraction was performed using a standardized data collection sheet before statistical analysis.

The following demographic and clinical variables were recorded:

- Age at operation;
- Sex;
- Type of thyroid surgery;
- Presence of multiple tumor foci;
- Presence of concomitant papillary thyroid carcinoma or papillary thyroid microcarcinoma, if reported;
- MEN syndrome status, if documented;
- RET mutation status, if documented in the medical record.

RET status was extracted as documented in the medical record. The available records did not consistently distinguish germline from somatic RET testing. Two RET-positive patients had documented MEN2A, whereas the testing context could not be verified reliably for the remaining RET-positive cases. RET status was therefore reported descriptively and was not included in the secretory-category models.

The following biochemical variables were recorded:

- Preoperative serum calcitonin;
- Preoperative serum CEA;
- Laboratory reporting status of calcitonin as detectable or undetectable;
- Assay lower reporting limit for calcitonin.

The following pathological variables were recorded:

- Dominant MTC tumor diameter in millimeters;
- Multifocality;
- Lymphovascular invasion;
- Perineural invasion;
- Capsular invasion;
- Tumor necrosis;
- Extrathyroidal extension;
- Pathological nodal assessment as pN0, pN-positive, or pNx;
- Total number of lymph nodes examined;
- Number of metastatic lymph nodes;
- Documented distant metastatic status;
- Presence of concomitant papillary thyroid carcinoma or other thyroid neoplasm.

The size of the largest lymph-node metastatic deposit, reliable subdivision into pN1a and pN1b, and complete pathological TNM stage were specifically sought during revision but were not systematically retrievable from the structured records. These variables were therefore not retrospectively reconstructed or imputed. The analysis was restricted to biochemical secretion metrics and pathological correlates relevant to the tumor diameter–adjusted secretory-category hypothesis.

### 2.6. Ancillary Diagnostic Variables and Data Completeness

Preoperative fine-needle aspiration cytology (FNAC), calcitonin measurement in fine-needle aspiration washout fluid (FNA-Ct), documented European Thyroid Imaging Reporting and Data System (EU-TIRADS) classification, standardized calcitonin immunohistochemical intensity or extent, and three-dimensional pathological tumor measurements were not performed or documented uniformly for all patients. A targeted retrospective review showed that these variables were available only in limited, clinically selected subsets and that the evaluable patients were not necessarily the same across variables.

These investigations had been performed according to individual clinical indication rather than a standardized study protocol. Consequently, their availability was substantially incomplete and indication-dependent. Further subdivision of these limited subsets into the serum-calcitonin-undetectable, hyposecretory, normosecretory, and hypersecretory groups would have produced sparse and potentially empty cells, unstable estimates, and a substantial risk of selection and verification bias.

These ancillary variables were therefore not included in category-specific inferential comparisons. Undocumented procedures were classified as unavailable rather than absent. No imputation, simulation, retrospective assignment of unreported immunostaining scores, or reconstruction of missing values was performed.

### 2.7. Biochemical Measurements

Preoperative serum calcitonin and CEA values were obtained from the institutional laboratory database. For each patient, the preoperative value used in analysis was defined as the last available serum measurement obtained before index thyroid surgery. Both calcitonin and CEA were measured as part of routine clinical evaluation of patients with suspected or confirmed MTC.

Serum calcitonin values were reported in pg/mL. The available historical laboratory export consistently preserved the measured result and the lower reporting limit of <2 pg/mL. However, the assay manufacturer and platform, analytical or functional sensitivity, and contemporaneous sex-specific reference intervals could not be verified reliably across the retrospective study period. These characteristics were therefore not retrospectively assigned or reported. Values below the reporting limit were treated as left-censored measurements rather than as true numeric values of 2 pg/mL. Patients with calcitonin reported as <2 pg/mL were excluded from the primary log-transformed continuous model but were retained as a separate descriptive subgroup.

CEA values were reported in ng/mL. CEA was used as a complementary biochemical marker and was analyzed descriptively across calcitonin secretory categories. No CEA-based outcome-derived cut-off was generated.

### 2.8. Pathological Assessment

All pathological variables were obtained from finalized institutional pathology reports signed out during routine clinical care. Final diagnoses were rendered according to the World Health Organization classification criteria in use at the time of routine reporting. No centralized slide re-review, retrospective reclassification according to a single WHO edition, or additional histopathological reinterpretation was performed for the present study. Pathological staging, when explicitly documented, followed the eighth edition of the American Joint Committee on Cancer TNM system. Complete pathological TNM stage was not consistently retrievable and was not retrospectively reconstructed. Tumor diameter was recorded in millimeters according to the final pathology report.

MTC diagnoses were accepted from the finalized institutional pathology reports. Case-level details of the immunohistochemical panels used during routine diagnosis were not consistently retrievable, including for the 17 patients with serum calcitonin below the assay reporting limit. Therefore, the specific use of calcitonin, CEA, chromogranin A, synaptophysin, or other markers could not be reported reliably for each patient, and diagnostic confirmation methods could not be compared between patients with detectable and undetectable serum calcitonin. No retrospective immunohistochemical scoring, reinterpretation, or assumption regarding unreported markers was performed.

For the primary analysis, the anatomical predictor was defined as the dominant MTC tumor diameter, corresponding to the largest medullary carcinoma focus reported in the specimen. In multifocal cases where more than one MTC focus was reported, the largest focus was used as the dominant tumor diameter to maintain consistency with conventional pathological diameter reporting. When multiple tumor foci were recorded as separate measurements, total MTC tumor burden was also calculated as the sum of all reported MTC focus diameters and used only for sensitivity analysis. Three orthogonal pathological tumor dimensions were available only in a small subset of patients. Therefore, reliable ellipsoid tumor volumes could not be calculated for the complete cohort or compared across the serum-calcitonin-undetectable subgroup and the three residual-based secretory categories. Tumor volume was not reconstructed from a single maximum diameter because assuming spherical tumor geometry would introduce unverifiable geometric error and pseudo-precision. FNA-Ct/MTC-volume ratios could not be standardized because FNA-Ct values were available only in a limited selected subset and the exact aspirated target, number of needle passes, and washout dilution volume were not consistently documented. Consequently, neither serum calcitonin/MTC-volume nor FNA-Ct/MTC-volume indices were generated.

Pathological nodal assessment was categorized as pN0 when at least one lymph node was examined and no metastatic lymph node was identified, pN-positive when at least one metastatic lymph node was identified, and pNx when no lymph node was pathologically examined. Because nodal-compartment information was not consistently retrievable, pN-positive cases were not routinely subdivided into pN1a and pN1b. Distant metastatic disease was categorized as documented M1 when explicitly recorded. The absence of documented distant metastasis was used only for the restricted sensitivity analysis and was not interpreted as pathological confirmation of pM0.

Micro-MTC was defined as medullary thyroid carcinoma with a dominant tumor diameter of ≤10 mm. The following adverse pathological features were evaluated individually:

- Lymphovascular invasion;
- Perineural invasion;
- Capsular invasion;
- Tumor necrosis;
- Extrathyroidal extension.

A composite adverse pathological outcome was defined as the presence of at least one of the following features: lymphovascular invasion, perineural invasion, capsular invasion, tumor necrosis, or extrathyroidal extension.

Lymphovascular invasion and perineural invasion were considered key invasive pathological features because they reflect tumor interaction with vascular, lymphatic, and neural structures and may indicate more aggressive local biological behavior independent of tumor diameter alone.

### 2.9. Definition of Calcitonin Secretory Density

As an initial descriptive measure of calcitonin secretion relative to dominant tumor diameter, calcitonin secretory density was calculated in patients with detectable calcitonin as:

Calcitonin secretory density = Preoperative serum calcitonin (pg/mL)/Dominant tumor diameter (mm)

This ratio was used as a simple supportive index of calcitonin secretion per millimeter of tumor diameter. However, because both calcitonin and dominant tumor diameter showed skewed distributions and because a simple ratio may be disproportionately influenced by very small tumors, the primary classification of secretory category was based on a log-linear size-adjusted residual model rather than on the raw ratio alone.

### 2.10. Construction of the Size-Adjusted Calcitonin Secretory Model

The primary objective of the analysis was to determine whether calcitonin secretion varied beyond what would be expected from tumor diameter alone. Because serum calcitonin values were right-skewed and dominant tumor diameter also showed a non-normal distribution, both variables were log-transformed before modeling.

In the detectable-calcitonin analytic cohort, the relationship between tumor diameter and calcitonin was modeled using the following log-linear regression equation:

log_10_ (calcitonin) = β0 + β1 × log_10_ (dominant tumor diameter)

For each patient, the expected log-transformed calcitonin value was calculated from this model based on tumor diameter. The size-adjusted calcitonin secretory residual was then defined as:

Secretory residual = log_10_ (observed calcitonin) − log_10_ (expected calcitonin based on tumor diameter)

A positive residual indicated that the observed calcitonin level was higher than expected for the patient’s tumor diameter. A negative residual indicated that the observed calcitonin level was lower than expected for the patient’s tumor diameter.

This residual was used as the primary continuous measure of tumor size–adjusted calcitonin secretion.

### 2.11. Classification of Exploratory Cohort-Derived Secretory Categories

Patients in the detectable-calcitonin analytic cohort were assigned to three exploratory, cohort-derived secretory categories according to tertiles of the size-adjusted calcitonin secretory residual:

1. Hyposecretory category: patients in the lowest tertile of the residual distribution, representing tumors with lower-than-expected calcitonin secretion for their diameter;
2. Normosecretory category: patients in the middle tertile, representing tumors with calcitonin secretion close to the expected range for their diameter;
3. Hypersecretory category: patients in the highest tertile, representing tumors with higher-than-expected calcitonin secretion for their diameter.

Tertile-based grouping was selected because no validated clinical cut-off exists for tumor size–adjusted calcitonin secretion. This approach avoided outcome-derived threshold selection and allowed evaluation of biological gradients in calcitonin secretion without generating a prognostic risk score. These tertile-based categories were specific to the present cohort and were not intended to represent externally validated biological subtypes or clinical decision thresholds.

Residual tertile cut-points were derived without reference to the pathological outcomes. A hypothesis-driven exploratory contrast compared the hypersecretory category with the combined non-hypersecretory categories because the biological question focused on disproportionately high calcitonin secretion relative to dominant tumor diameter. This contrast was not used to derive the residual thresholds and was not externally preregistered. Global three-group comparisons were also reported to show the complete distribution of pathological features across the cohort-derived categories.

### 2.12. Definition of Study Outcomes

The primary pathological outcome was the presence of invasive pathological features associated with the calcitonin secretory category.

The main individual pathological outcomes were:

- Lymphovascular invasion;
- Perineural invasion.

Secondary pathological outcomes were:

- Capsular invasion;
- Tumor necrosis;
- Extrathyroidal extension;
- Composite adverse pathological outcome.

No outcome-derived thresholds, ROC-derived cut-offs, prognostic scores, recurrence prediction models, or nodal prediction models were generated.

### 2.13. Handling of Undetectable Calcitonin Values

Patients with calcitonin values reported as <2 pg/mL were handled separately because these values represented left-censored laboratory results below the lower reporting limit of the assay. Assigning an arbitrary numeric value to these patients could distort log-transformed modeling and artificially inflate the hyposecretory group.

Therefore, the following approach was used:

1. Patients with calcitonin <2 pg/mL were included in the overall cohort description;
2. They were excluded from the primary continuous calcitonin residual model;
3. They were summarized separately as a serum-calcitonin-undetectable subgroup;
4. Their demographic, biochemical, and pathological characteristics were descriptively compared with the detectable-calcitonin cohort;
5. No imputed calcitonin value was used in the primary analysis.

This strategy was chosen to preserve biological information from MTC cases with serum calcitonin below the assay reporting limit while avoiding invalid continuous modeling of censored biochemical values.

### 2.14. Sensitivity Analyses

Several sensitivity analyses were conducted to assess the robustness of the findings.

First, the size-adjusted calcitonin residual model was repeated using total MTC tumor burden instead of dominant tumor diameter in multifocal cases when separate tumor focus diameters were available.

Second, patients with micro-MTC were evaluated descriptively to determine whether very small tumors disproportionately influenced calcitonin secretory density or residual-based category assignment.

Third, patients with concomitant papillary thyroid carcinoma or papillary thyroid microcarcinoma were assessed descriptively to determine whether the presence of an additional thyroid malignancy altered the interpretation of calcitonin-based MTC secretory categories.

Fourth, the serum-calcitonin-undetectable subgroup was summarized separately to determine whether this group represented a distinct low-volume and low-invasive pathological profile.

Fifth, a sex-adjusted secretion model was constructed in the 53 patients with detectable calcitonin:

log_10_ (calcitonin) = β0 + β1 × log_10_ (dominant tumor diameter) + β2 × male sex.

Sex-adjusted residuals were divided into tertiles using the same cohort-derived approach. Agreement with the primary category assignment was assessed using raw percentage agreement and Cohen’s kappa.

Sixth, among patients with pathological lymph-node assessment, a metastatic-burden-adjusted model was constructed:

log_10_ (calcitonin) = β0 + β1 × log_10_ (dominant tumor diameter) + β2 × male sex + β3 × log(1 + number of metastatic lymph nodes) + β4 × documented M1 status.

Residual-based categories from this model were compared with the primary categories using raw percentage agreement and Cohen’s kappa. Seventh, the relationship between dominant tumor diameter and preoperative calcitonin was re-estimated in patients with pN0 disease and no documented distant metastasis. Because invasive pathological events were sparse in this restricted subgroup, no secretory-category versus pathology comparison was performed.

### 2.15. Statistical Analysis

Primary statistical analyses were performed using IBM SPSS Statistics version 22.0 (IBM Corp., Armonk, NY, USA). Additional linear regression sensitivity analyses and category-concordance calculations were performed using Python version 3.13.5 with statsmodels version 0.14.6, SciPy version 1.17.0, and scikit-learn version 1.8.0. Continuous variables were assessed for distributional normality using the Shapiro–Wilk test and visual inspection of histograms and Q–Q plots. Nominal two-sided *p* values < 0.05 were interpreted as unadjusted evidence of association, whereas FDR-adjusted q values < 0.05 were considered statistically significant after multiplicity adjustment. Effect sizes, confidence intervals, directionality, and the exploratory nature of the analyses were emphasized throughout.

Continuous variables were expressed as median and interquartile range or median and range, depending on distribution and reporting requirements. Categorical variables were expressed as number and percentage.

Comparisons between two groups were performed using:

- Mann–Whitney U test for non-normally distributed continuous variables;
- Student’s *t*-test for normally distributed continuous variables;
- Chi-square test for categorical variables when expected cell counts were adequate;
- Fisher’s exact test for categorical variables with small expected cell counts.

For the hypothesis-driven exploratory hypersecretory versus non-hypersecretory contrast, unadjusted odds ratios with 95% confidence intervals were calculated, and Fisher’s exact *p* values were reported as nominal results. Benjamini–Hochberg false-discovery-rate-adjusted q values were calculated across the three pathological outcomes reported in the primary contrast as an exploratory multiplicity assessment. Comparisons among the three calcitonin secretory categories were performed using:

- Kruskal–Wallis test for continuous variables;
- Chi-square test or Fisher’s exact test for categorical variables.

The relationship between preoperative calcitonin and tumor diameter was first evaluated using Spearman rank correlation. A log-linear regression model was then constructed using log-transformed calcitonin and log-transformed dominant tumor diameter to derive size-adjusted calcitonin secretory residuals. Additional linear regression models incorporated sex, log-transformed metastatic lymph-node count as log(1 + count), and documented M1 status as described in the sensitivity analyses. Category-assignment agreement was quantified using raw percentage agreement and Cohen’s kappa.

Associations between secretory category and pathological features were evaluated using group comparisons and logistic regression where appropriate. Because of the limited sample size and the exploratory nature of the study, multivariable models were deliberately restricted to avoid overfitting. When regression models were used, the number of covariates was limited according to the number of observed events. The main explanatory variable was hypersecretory category status. Tumor diameter was not routinely re-entered into models based on the residual category because category assignment was already adjusted for dominant tumor diameter.

No ROC curve analysis was performed, and no outcome-derived cut-off values were generated.

### 2.16. Data Quality and Missing Data Management

Before analysis, the dataset was checked for duplicate patient-level records, missing values, out-of-range values, and implausible biochemical or pathological entries. Patient-level duplication was resolved before construction of the final cohort.

Patients missing either preoperative calcitonin or pathological tumor diameter were not eligible for category modeling. No statistical imputation was performed for missing biochemical or pathological data. Analyses were conducted using available complete data for each variable.

For calcitonin values below the assay reporting limit, no artificial numeric replacement was used in the primary analysis. These patients were treated as having undetectable calcitonin and were handled as a separate subgroup as described above. Ancillary diagnostic variables with indication-dependent missingness were not subjected to complete-case inferential analysis because the probability of data availability was related to clinical suspicion and diagnostic work-up. Such analyses would therefore not represent the complete cohort and could produce biased category-specific estimates. Missing ancillary information was not interpreted as evidence that the corresponding diagnostic procedure had not been performed.

### 2.17. Rationale for Methodological Approach

The central methodological principle of the study was to separate tumor burden from tumor secretory behavior. Absolute calcitonin values are strongly influenced by tumor size; therefore, direct comparison of raw calcitonin values may primarily reflect anatomical tumor burden. By modeling calcitonin as a function of tumor diameter and using the residual from this model, the study aimed to identify patients whose calcitonin secretion was higher or lower than expected for their tumor size.

This approach assigned patients to exploratory hyposecretory, normosecretory, and hypersecretory categories without relying on arbitrary clinical thresholds or outcome-derived cut-offs. These cohort-derived categories were then compared with invasive pathological features to explore whether dominant-tumor-diameter–adjusted calcitonin secretion showed clinically relevant pathological patterns.

### 2.18. Use of Generative Artificial Intelligence

Generative artificial intelligence tools provided by OpenAI, including ChatGPT (GPT-5; OpenAI, San Francisco, CA, USA; accessed in August 2026) including its image-generation functionality, were used during manuscript preparation for language editing, drafting and organizational assistance, and preparation of the initial graphical-abstract layout. No synthetic patient data were generated. All patient-level data, numerical results, references, interpretations, and final visual content were independently reviewed and approved by the authors.

## 3. Results

### 3.1. Study Cohort and Calcitonin Detectability

**Figure 1:**
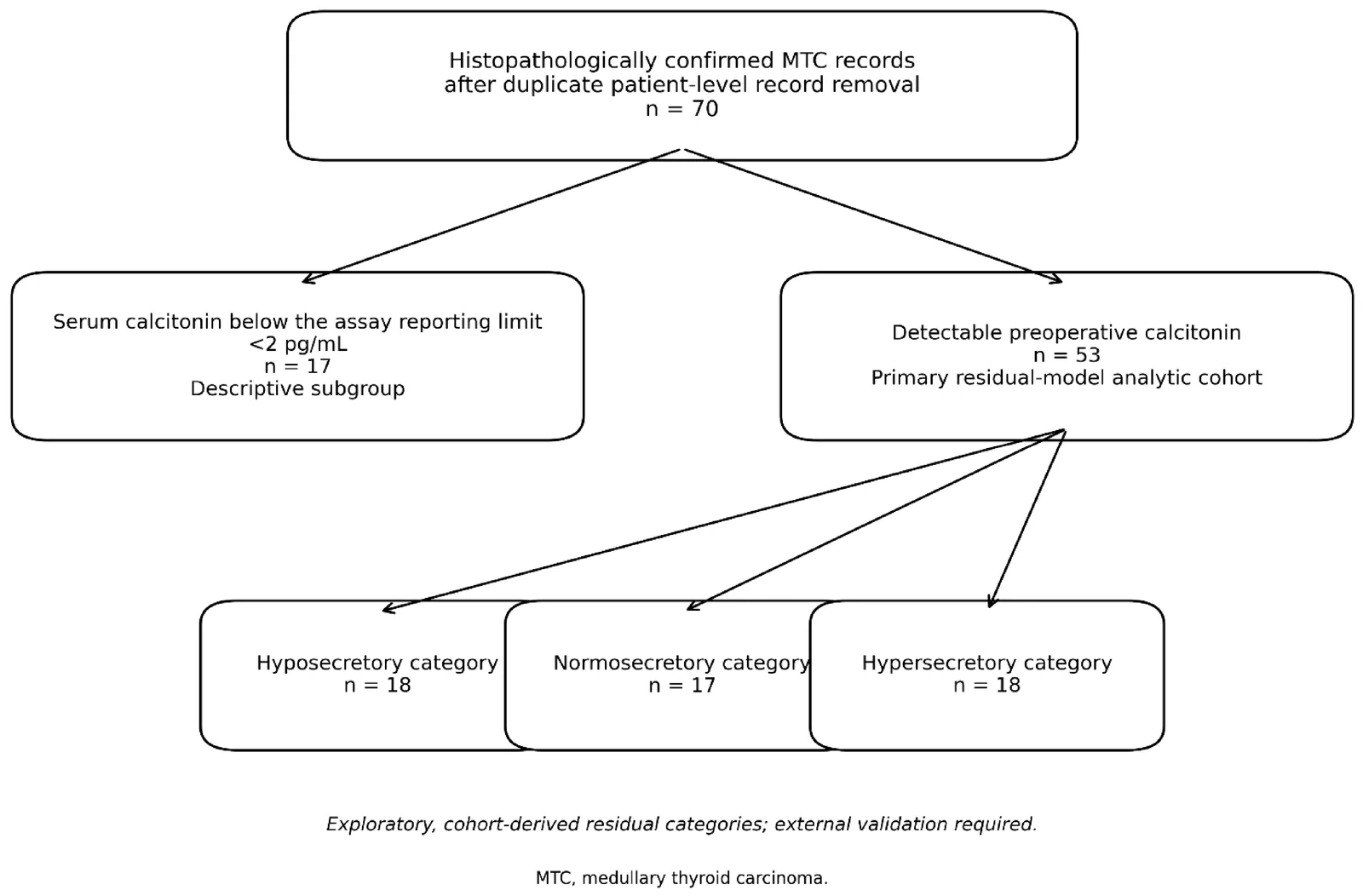
Study flow and analytic cohort allocation. Seventy unique patients with histopathologically confirmed medullary thyroid carcinoma were included after duplicate patient-level records were removed. Seventeen patients with preoperative serum calcitonin below the institutional laboratory reporting limit (<2 pg/mL) were retained as a serum-calcitonin-undetectable descriptive subgroup. The primary size-adjusted calcitonin secretory category analysis was performed in 53 patients with detectable calcitonin.

A total of 70 unique patients with histopathologically confirmed medullary thyroid carcinoma were included in the source cohort. Of these, 17 patients (24.3%) had preoperative serum calcitonin below the institutional reporting limit (<2 pg/mL) and were described separately. The remaining 53 patients (75.7%) had detectable preoperative calcitonin values and constituted the primary analytic cohort for size-adjusted calcitonin secretory category analysis (Figure 1).

Sixty-nine patients underwent total thyroidectomy, and one underwent right thyroid lobectomy. Central and/or lateral compartment neck dissection was performed in 45 patients according to clinical indication. At least one lymph node was retrieved and examined pathologically in 58 patients.

The baseline characteristics of the overall cohort according to calcitonin detectability are summarized in Table 1. The median age of the overall cohort was 55.5 years. Patients with serum calcitonin below the assay reporting limit had significantly smaller dominant tumor diameters than patients with detectable calcitonin [median, 4.0 mm vs. 12.0 mm; *p* < 0.001]. Similarly, the serum-calcitonin-undetectable subgroup had lower preoperative CEA levels [median, 1.5 ng/mL vs. 3.2 ng/mL; *p* < 0.001]. Dominant tumor diameter ≤10 mm was more frequent among patients with serum calcitonin below the assay reporting limit [82.4% vs. 41.5%; *p* = 0.005]. Concomitant papillary thyroid lesions were also more frequent in the serum-calcitonin-undetectable subgroup (58.8% vs. 26.4%; *p* = 0.020). Invasive pathological features were numerically less frequent in the serum-calcitonin-undetectable subgroup, although differences in lymphovascular invasion, perineural invasion, capsular invasion, extrathyroidal extension, tumor necrosis, and composite adverse pathological outcome did not reach statistical significance.

### 3.2. Availability of Ancillary Diagnostic Data

A targeted review of the available cytology, laboratory, ultrasound, and pathology records confirmed that preoperative FNAC, FNA-Ct, documented EU-TIRADS classification, standardized quantitative or semiquantitative calcitonin immunohistochemistry, and complete three-dimensional pathological tumor measurements were available only in small and clinically selected subsets. Documentation for these variables was limited and substantially incomplete across the 70-patient cohort, and the evaluable cases were not identical across variables.

After subdivision into the serum-calcitonin-undetectable, hyposecretory, normosecretory, and hypersecretory groups, the number of evaluable patients per cell was insufficient for reliable category-specific estimates. No comparative *p* values, diagnostic-performance estimates, or regression models were therefore generated for these ancillary variables. The available documentation was also insufficient to determine reliably whether FNA-Ct alone established the diagnosis in any patient with serum calcitonin below the assay reporting limit.

### 3.3. Relationship Between Tumor Diameter and Preoperative Calcitonin

In the detectable-calcitonin cohort, preoperative calcitonin showed a significant positive correlation with dominant tumor diameter (Spearman ρ = 0.644, *p* < 0.001). The association remained similar when total MTC tumor burden was used instead of dominant tumor diameter (Spearman ρ = 0.657, *p* < 0.001).

A log-linear model was constructed to estimate expected calcitonin secretion according to tumor diameter:

log_10_ (calcitonin) = 0.383 + 1.712 × log_10_ (dominant tumor diameter)

**Figure 2:**
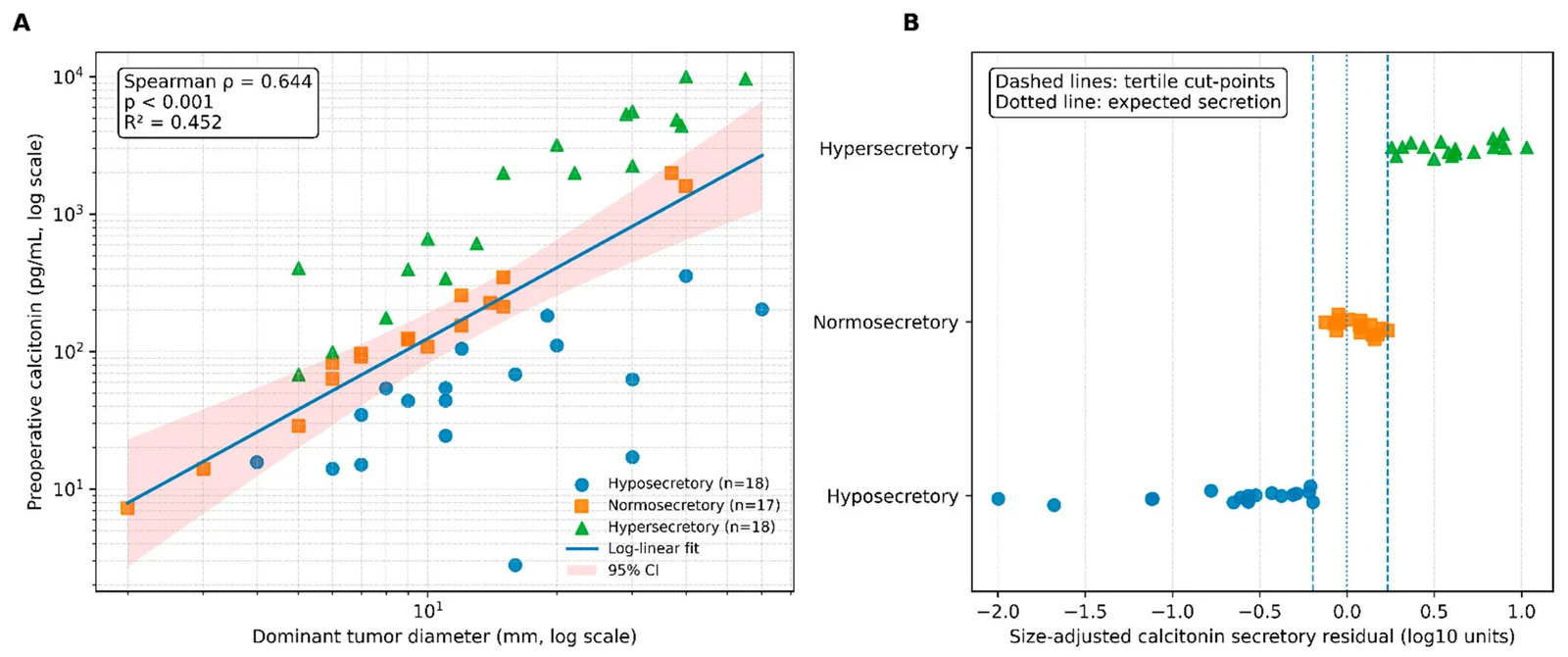
Tumor diameter–adjusted calcitonin secretion model and residual-based phenotype definition. (**Panel A**) shows the log-linear relationship between dominant tumor diameter and preoperative calcitonin in patients with detectable calcitonin. The fitted model explained 45.2% of calcitonin variability. (**Panel B**) shows the distribution of size-adjusted calcitonin secretory residuals used to define hyposecretory, normosecretory, and hypersecretory phenotypes. Dashed lines indicate tertile cut-points; the dotted line indicates expected secretion.

Dominant tumor diameter explained 45.2% of the variance in log-transformed calcitonin levels (R^2^ = 0.452; adjusted R^2^ = 0.442; *p* < 0.001) (Table 2, Figure 2). The remaining 54.8% of calcitonin variability was not explained by dominant tumor diameter. This unexplained variation may reflect tumor-intrinsic secretory differences, sex-related variation, assay variability, nodal or distant metastatic burden, and other unmeasured clinical or pathological factors.

**Figure 3:**
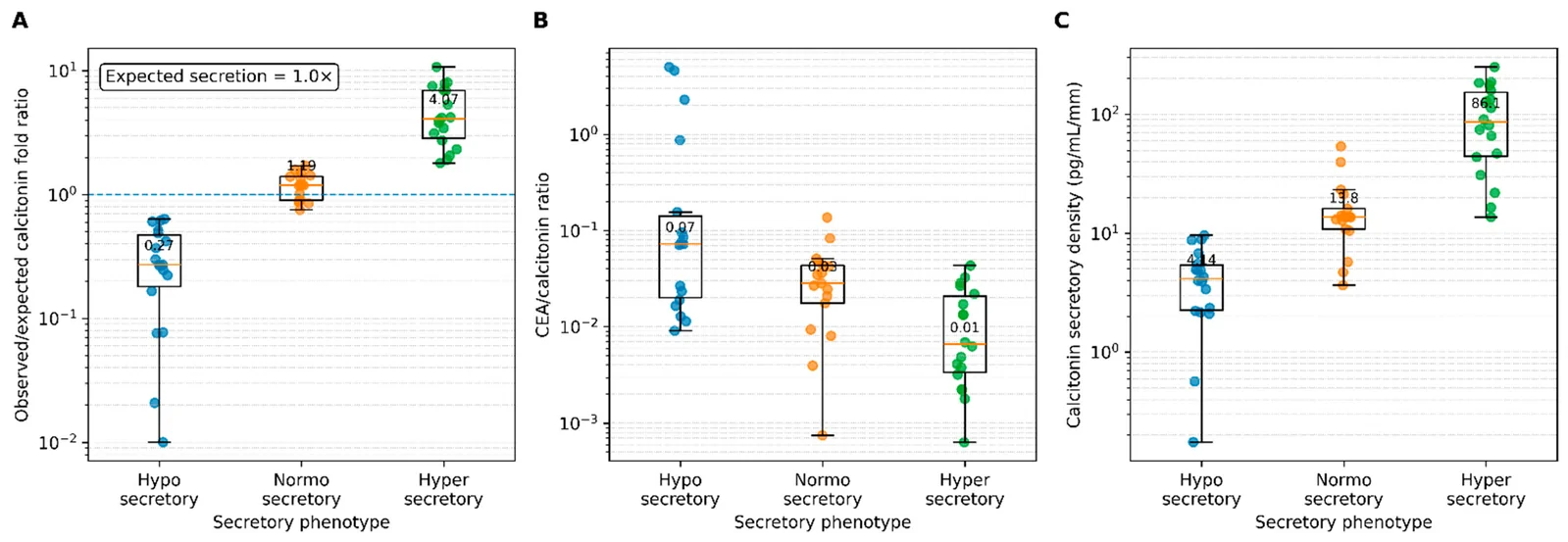
Biochemical gradients across tumor size–adjusted calcitonin secretory phenotypes. (**Panel A**) shows the observed-to-expected calcitonin fold ratio derived from the residual model. (**Panel B**) shows the CEA/calcitonin ratio, which was highest in the hyposecretory phenotype. (**Panel C**) shows calcitonin secretory density. Boxes indicate interquartile ranges, horizontal lines indicate medians, and points represent individual patients.

For each patient, a size-adjusted calcitonin secretory residual was calculated as the difference between observed and tumor diameter–predicted log-transformed calcitonin. Patients were then assigned to three exploratory, cohort-derived residual categories: hyposecretory (*n* = 18), normosecretory (*n* = 17), and hypersecretory (*n* = 18). The median observed-to-expected calcitonin fold ratio was 0.27 in the hyposecretory group, 1.19 in the normosecretory group, and 4.07 in the hypersecretory group (Table 2, Figure 3).

### 3.4. Nodal and Distant Metastatic Burden

Among the 53 patients in the detectable-calcitonin analytic cohort, 24 were pN0, 23 were pN-positive, and 6 were pNx. Eight patients had documented distant metastatic disease. The distribution of pN0, pN-positive, and pNx status did not differ among the hyposecretory, normosecretory, and hypersecretory categories (global *p* = 0.934). The number of metastatic lymph nodes also did not differ significantly among the categories (*p* = 0.269), and the distribution of documented M1 disease did not differ significantly among the categories (*p* = 0.108).

The total number of lymph nodes examined differed among the categories (*p* = 0.008), reflecting differences in the extent of clinically indicated neck dissection rather than an intrinsic measure of metastatic burden. The size of the largest lymph-node metastatic deposit, reliable pN1a/pN1b subdivision, and complete pathological TNM stage were not systematically retrievable and were not analyzed. Detailed data are presented in Supplementary Table S5.

### 3.5. Biochemical and Dominant-Tumor-Diameter Characteristics Across Secretory Categories

Clinical and biochemical characteristics according to secretory category are shown in Supplementary Table S1. Age did not differ significantly among the three groups (*p* = 0.282). Female sex was less frequent in the hypersecretory category compared with the hyposecretory and normosecretory groups [38.9% vs. 72.2% and 76.5%, respectively; *p* = 0.049].

By design, preoperative calcitonin differed significantly across the three categories. Median calcitonin values were 49.1 pg/mL in the hyposecretory group, 123.0 pg/mL in the normosecretory group, and 2000.0 pg/mL in the hypersecretory group (*p* < 0.001). Preoperative CEA also differed significantly across categories, with the highest median CEA level observed in the hypersecretory group [5.8 ng/mL vs. 2.6 ng/mL and 2.6 ng/mL; *p* = 0.041].

Dominant tumor diameter was numerically larger in the hypersecretory group [median, 17.5 mm] than in the hyposecretory [11.5 mm] and normosecretory [9.0 mm] groups, but this difference did not reach statistical significance (*p* = 0.127). Total MTC tumor burden showed a similar pattern (*p* = 0.148). Calcitonin secretory density increased markedly from the hyposecretory to hypersecretory category [median, 4.1, 13.8, and 86.1 pg/mL/mm, respectively; *p* < 0.001].

The CEA/calcitonin ratio demonstrated an inverse gradient across categories. The ratio was highest in the hyposecretory group and lowest in the hypersecretory group [median, 0.073, 0.028, and 0.007, respectively; *p* < 0.001], indicating that low calcitonin secretion relative to tumor size may be accompanied by relatively greater CEA predominance (Supplementary Table S1, Figure 3).

### 3.6. Pathological Features According to Secretory Category

Pathological characteristics across the three secretory categories are presented in Supplementary Table S2. Lymphovascular invasion was observed in 5/18 patients (27.8%) in the hyposecretory group, 4/17 patients (23.5%) in the normosecretory group, and 10/18 patients (55.6%) in the hypersecretory group. Although the global three-group comparison did not reach statistical significance (*p* = 0.135), lymphovascular invasion was numerically most frequent in the hypersecretory category.

A similar pattern was observed for perineural invasion. Perineural invasion was present in 2/18 patients (11.1%) in the hyposecretory group, 1/17 patient (5.9%) in the normosecretory group, and 6/18 patients (33.3%) in the hypersecretory group (global *p* = 0.117). Capsular invasion, tumor necrosis, and extrathyroidal extension did not differ significantly across categories.

The composite adverse pathological outcome was present in 6/18 patients (33.3%) in the hyposecretory group, 5/17 patients (29.4%) in the normosecretory group, and 10/18 patients (55.6%) in the hypersecretory group (*p* = 0.264).

### 3.7. Hypersecretory Category and Invasive Pathological Features

Because the biological hypothesis focused on disproportionately high calcitonin secretion, a hypothesis-driven exploratory comparison was performed between the cohort-derived hypersecretory category and the combined non-hypersecretory categories. Residual tertile thresholds had been defined independently of the pathological outcomes.

**Figure 4:**
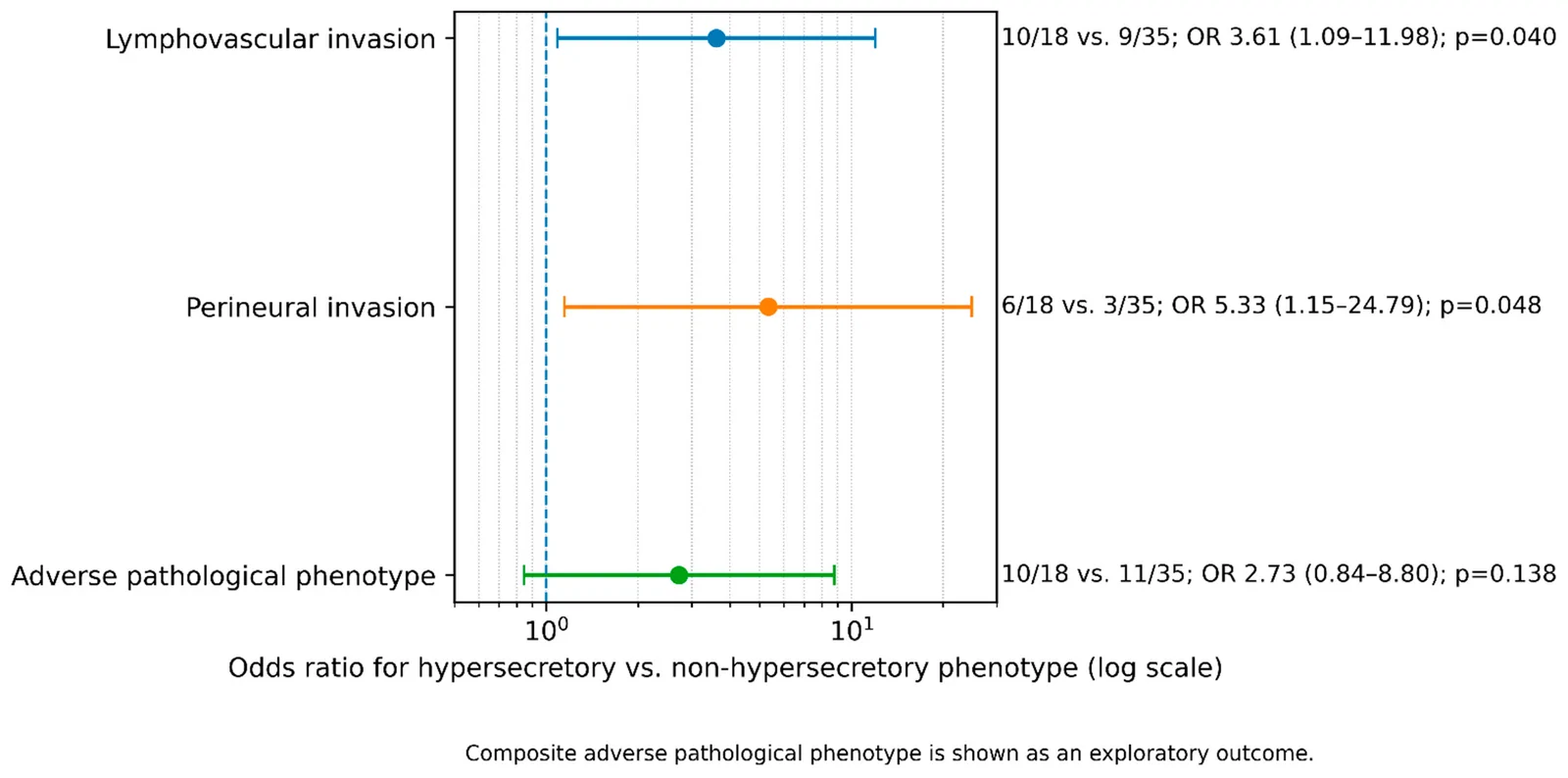
Hypersecretory phenotype and invasive pathological features. Forest plot showing unadjusted odds ratios for invasive pathological features in hypersecretory versus non-hypersecretory tumors. Lymphovascular and perineural invasion showed nominally significant enrichment in the hypersecretory phenotype. Composite adverse pathological phenotype is shown as an exploratory outcome.

In this exploratory binary contrast, lymphovascular invasion [55.6% vs. 25.7%; OR 3.61, 95% CI 1.09–11.98; nominal Fisher’s exact *p* = 0.040; FDR q = 0.072] and perineural invasion [33.3% vs. 8.6%; OR 5.33, 95% CI 1.15–24.79; nominal Fisher’s exact *p* = 0.048; FDR q = 0.072] were more frequent in the hypersecretory category than in the combined non-hypersecretory categories (Table 3, Figure 4). However, the global three-group comparisons were not statistically significant for lymphovascular invasion (*p* = 0.135) or perineural invasion (*p* = 0.117). These findings were therefore interpreted as exploratory and hypothesis-generating.

Exploratory age- and sex-adjusted logistic models yielded directionally consistent estimates for lymphovascular invasion and perineural invasion, but these models were interpreted cautiously because of limited event numbers and are reported in Supplementary Table S4.

### 3.8. Sensitivity Analysis Using Total MTC Tumor Burden

Sensitivity analysis using total MTC tumor burden instead of dominant tumor diameter produced similar results (Supplementary Table S3). The log-linear model using total tumor burden explained 47.1% of calcitonin variability (R^2^ = 0.471; adjusted R^2^ = 0.461; *p* < 0.001), slightly higher than the dominant-diameter model. Category assignment was highly concordant between the dominant-diameter and total-tumor-burden models. Overall concordance was 51/53 patients (96.2%), with a Cohen’s κ of 0.943. All patients classified as hypersecretory by the dominant-diameter model remained hypersecretory when total MTC tumor burden was used. This high concordance suggests that the hypersecretory category was not materially dependent on whether tumor burden was represented by dominant diameter or summed multifocal tumor burden.

### 3.9. Sensitivity Analysis Accounting for Sex

Adding sex to the dominant-tumor-diameter calcitonin model increased the explained variance from R^2^ = 0.452 to R^2^ = 0.482 (adjusted R^2^ = 0.461). Dominant tumor diameter remained associated with preoperative calcitonin [β = 1.595, 95% CI 1.057–2.134; *p* < 0.001]. Male sex was associated with an estimated 0.312-log10-unit increase in calcitonin, although the confidence interval included the null [95% CI −0.057 to 0.681; *p* = 0.096].

Agreement between the primary and sex-adjusted residual categories was 45/53 patients (84.9%), with a Cohen’s kappa of 0.774. Sixteen of the 18 patients originally assigned to the hypersecretory category remained hypersecretory after sex adjustment. These results are presented in Supplementary Table S6.

### 3.10. Sensitivity Analyses Accounting for Nodal and Distant Metastatic Burden

Forty-seven patients in the detectable-calcitonin cohort had pathological lymph-node assessment. In a model incorporating dominant tumor diameter, sex, log-transformed metastatic lymph-node count, and documented M1 status, dominant tumor diameter remained independently associated with calcitonin [β = 1.447, 95% CI 0.734–2.161; *p* < 0.001]. The model explained 48.3% of calcitonin variability (adjusted R^2^ = 0.434). Neither metastatic lymph-node count (*p* = 0.122) nor documented M1 status (*p* = 0.241) was independently associated with calcitonin in this event-limited model.

Agreement between the primary and metastatic-burden-adjusted residual categories was 40/47 patients (85.1%), with a Cohen’s kappa of 0.776.

In a separate sensitivity analysis restricted to 23 patients with pN0 disease and no documented distant metastasis, dominant tumor diameter remained strongly associated with preoperative calcitonin [β = 1.912, 95% CI 1.160–2.664; *p* < 0.001], with R^2^ = 0.571 (adjusted R^2^ = 0.550). Only two lymphovascular invasion events and no perineural invasion events occurred in this restricted subgroup; therefore, no secretory-category versus pathology comparison was performed. Detailed findings are presented in Supplementary Table S7.

## 4. Discussion

The present study introduces an exploratory, cohort-derived framework for interpreting calcitonin secretion relative to dominant tumor diameter in MTC. The primary model showed that dominant tumor diameter explained 45.2% of circulating calcitonin variability. In a hypothesis-driven exploratory contrast, the hypersecretory category showed nominal enrichment for lymphovascular and perineural invasion; however, the global three-group comparisons were not statistically significant, and both associations had FDR-adjusted q values of 0.072. These results should therefore be considered hypothesis-generating rather than evidence that the cohort-derived categories represent established biological MTC phenotypes.

Circulating calcitonin may originate not only from the dominant thyroid tumor but also from metastatic lymph-node deposits and distant metastases. This represents an important potential limitation of a primary-tumor-diameter model. Nevertheless, dominant tumor diameter remained independently associated with calcitonin after adjustment for sex, metastatic lymph-node count, and documented M1 status. The relationship was also preserved in patients with pN0 disease and no documented distant metastasis, in whom dominant tumor diameter explained 57.1% of calcitonin variability. These sensitivity analyses reduce, but do not eliminate, the possibility that the primary findings were driven by metastatic disease burden. Reliable pN1a/pN1b subdivision, the size of the largest nodal metastatic deposit, and complete pTNM stage were not systematically available.

Sex is another potential source of variation. Physiological basal calcitonin concentrations are generally higher in men, and the primary cohort-derived hypersecretory category contained a higher proportion of male patients. In the sex-adjusted secretion model, the estimated male coefficient was positive but did not reach conventional statistical significance. Residual-category assignment nevertheless remained substantially concordant with the primary model, and 16 of 18 originally hypersecretory patients remained in that category. These findings suggest that sex contributed to, but did not fully explain, the primary classification. Future validation studies should use assay-specific, sex-adjusted reference models.

This finding addresses a clinically familiar but insufficiently formalized problem in MTC: calcitonin is strongly associated with tumor burden at the population level, yet individual patients with tumors of comparable size may show markedly different biochemical profiles. Large clinical series have consistently demonstrated that calcitonin, tumor stage, nodal involvement, distant metastasis, and biochemical cure are interrelated determinants of outcome [2,7,10,11,12,13,20,21,22]. However, these studies generally evaluate calcitonin as an absolute value, a kinetic marker, or a threshold-based predictor. Such approaches are clinically useful, but they do not separate the amount of tumor from the secretory behavior of that tumor. In contrast, the residual-based model used in this study asks a different biological question: for a given tumor diameter, does the tumor secrete more or less calcitonin than expected? This shift from absolute biomarker interpretation to size-adjusted secretory profiling is the conceptual core of the present analysis.

The distinction is important because threshold-based calcitonin interpretation and residual-based secretory categorization are not interchangeable. Preoperative calcitonin thresholds have been widely studied in relation to nodal disease, extent of lymph node metastasis, and surgical decision-making [10,23,24]. More recent prediction models and nomogram-based strategies have also attempted to combine biochemical and tumor-related variables to predict lateral lymph node metastasis or distant disease [25,26]. These approaches answer an anatomical risk question: “How likely is disease spread?” The present analysis addresses a different question: “Does the tumor show discordant biochemical behavior after accounting for its size?” Therefore, size-adjusted calcitonin secretion should not be interpreted as a competing risk model. It is better understood as an exploratory analytical layer that may help explain why the same tumor diameter can generate different biochemical signatures.

European Thyroid Imaging Reporting and Data System categories 3–5 stratify the general ultrasound risk of malignancy but are not specific for MTC [27]. FNAC has limited sensitivity for MTC because of heterogeneous cytomorphology [28], whereas calcitonin measurement in FNA washout fluid may provide complementary diagnostic information [29], particularly in nodules with serum calcitonin concentrations of 10–100 pg/mL and inconclusive cytology [30,31]. In the present cohort, FNAC, FNA-Ct, and EU-TIRADS data were available only in small, clinically selected subsets and could not be compared reliably across the exploratory cohort-derived categories. Their potential diagnostic value should be evaluated prospectively using standardized cytology, washout protocols, ultrasound classification, and assay-specific thresholds.

The higher frequencies of lymphovascular and perineural invasion in the hypersecretory category provide a hypothesis-generating pathological signal. However, this interpretation requires caution: the global three-category comparisons were not statistically significant, and the nominal binary associations did not meet the FDR-adjusted significance threshold. Therefore, the present findings do not establish an invasive biological category. Rather, they support prospective investigation of whether calcitonin secretion that is disproportionately high relative to dominant tumor diameter is associated with microscopic routes of invasion.

This interpretation is compatible with the evolving pathological understanding of MTC. Contemporary grading frameworks emphasize that tumor biology cannot be inferred from diameter alone. The International Medullary Thyroid Carcinoma Grading System defines high-grade disease by a mitotic index of ≥5 per 2 mm^2^, a Ki-67 proliferative index of ≥5%, and/or the presence of tumor necrosis [16,17]. Ki-67 and mitotic count were not available in a standardized manner in the present cohort, and the current residual categories cannot replace formal histological grading. The observed nominal pattern instead supports prospective investigation of whether tumor diameter–adjusted calcitonin secretion parallels specific invasive pathological features.

The plausibility of secretory heterogeneity is supported by the known molecular and neuroendocrine diversity of MTC. MTC exists along a spectrum defined by germline or somatic RET activation, RAS-pathway alterations, neuroendocrine differentiation, peptide processing, and variable expression of calcitonin and CEA [1,3,6,8,32,33,34]. Modern targeted therapies directed against RET-altered disease further illustrate the biological importance of molecular subclassification in advanced MTC [35,36]. Although the present study was not designed to evaluate genotype–phenotype associations, future studies integrating tumor diameter–adjusted calcitonin residuals with RET/RAS status, transcriptomic profiling, calcitonin immunohistochemistry, CEA expression, Ki-67, and tumor grade could determine whether the cohort-derived secretory categories correspond to reproducible biological subtypes.

The serum-calcitonin-undetectable subgroup requires particular diagnostic caution. Serum calcitonin below the assay reporting limit should not automatically be equated with biologically non-secretory MTC, because circulating secretion, intracellular peptide synthesis, tissue immunoreactivity, and local release into FNA washout fluid represent related but non-equivalent processes. Potential preanalytical and analytical explanations should also be considered [5].

In the present cohort, the final diagnosis of MTC in the 17 patients with serum calcitonin below the reporting limit was accepted as documented in finalized institutional pathology reports. However, the case-level immunohistochemical panels used during routine diagnosis could not be reconstructed reliably from the available retrospective records. Consequently, we could not determine which specific markers had been used in each case or whether the diagnostic confirmation process differed from that in patients with detectable serum calcitonin. No retrospective assumption, reinterpretation, or reconstruction of unreported immunohistochemical data was performed.

Patients in this subgroup had smaller tumors and lower CEA levels, while invasive pathological features were numerically less frequent. The higher frequency of concomitant papillary thyroid lesions suggests that some MTCs may have been incidentally identified during surgery for another thyroid pathology. Accordingly, these patients are more accurately described as a serum-calcitonin-undetectable subgroup rather than as having calcitonin-negative or biologically non-secretory MTC. The available records were also insufficient to determine whether FNA-Ct was the sole diagnostic pathway in any patient in this subgroup.

The CEA/calcitonin relationship adds another layer to the interpretation of secretory heterogeneity. In this cohort, CEA levels were highest in the hypersecretory group, whereas the CEA/calcitonin ratio was highest in the hyposecretory group. This pattern suggests that calcitonin and CEA may reflect partially distinct aspects of tumor biology. Prior work has emphasized the complementary value of CEA, particularly when CEA and calcitonin kinetics diverge [6,7,8]. A relatively high CEA/calcitonin ratio in hyposecretory tumors may indicate a more CEA-dominant biochemical profile, reduced calcitonin secretion relative to anatomical tumor burden, or altered neuroendocrine differentiation. These possibilities remain speculative, but they are clinically relevant because surveillance strategies that rely heavily on calcitonin may be less informative in patients with low or discordant calcitonin secretion.

A methodological strength of the present study is the decision not to derive outcome-based thresholds. In small retrospective cohorts of rare malignancies, ROC-derived cut-offs and multivariable prediction models may appear attractive but are prone to overfitting, optimism, and poor transportability. Statistical literature has repeatedly cautioned that event-limited datasets require parsimonious modeling and careful interpretation of multivariable estimates [37,38,39]. For this reason, our primary approach was not to create a prognostic score but to define an exploratory analytical category using a dominant-tumor-diameter–adjusted residual. The use of residual tertiles avoided outcome-derived threshold selection, but it also produced categories that are inherently cohort-dependent. These categories should therefore be regarded as exploratory analytical labels rather than established biological phenotypes or clinical thresholds. Their reproducibility, calibration, and biological interpretation require evaluation in independent cohorts. Similarly, the restricted exploratory regression models were designed to test whether the direction and magnitude of association persisted after minimal adjustment, not to build a definitive predictive model.

The sensitivity analysis using total MTC tumor burden provides additional reassurance. Dominant tumor diameter is the conventional pathological size measure, but it may underestimate total C-cell tumor burden in multifocal disease. Repeating the model with total tumor burden produced a similar explained variance and very high concordance in category assignment. Importantly, all patients classified as hypersecretory using the dominant-diameter model remained hypersecretory when total tumor burden was used. This stability argues against the hypersecretory category being an artifact of how tumor size was operationalized. It also supports the practical use of dominant tumor diameter when total tumor burden cannot be reconstructed reliably from pathology reports.

Normalization to three-dimensional MTC volume could theoretically refine the distinction between anatomical tumor burden and secretory behavior. However, reliable pathological volume calculation requires three orthogonal measurements for each tumor focus and becomes particularly complex in irregular or multifocal tumors. These measurements were available only in a small selected subset of the present cohort. Estimating tumor volume from a single maximum diameter under a spherical assumption would introduce potentially substantial geometric error and pseudo-precision.

FNA-Ct/MTC-volume ratios are additionally influenced by the aspirated target, the number of needle passes, the amount of washout solution, and the assay platform. These procedural details were not standardized or consistently documented in our retrospective records. The high concordance between the dominant-diameter and summed-focus-diameter sensitivity models suggests that the identified category was not materially dependent on the available operational definition of tumor burden, although prospective validation using standardized three-dimensional volumetry remains warranted.

The clinical implications should be framed cautiously. Size-adjusted calcitonin secretion is not intended to dictate the extent of surgery, replace anatomical staging, substitute for RET testing, or override postoperative dynamic risk stratification [2,4,18,19]. Its value is interpretive rather than prescriptive. Whether disproportionately high calcitonin secretion relative to dominant tumor diameter should influence imaging, pathological assessment, or surveillance requires prospective external validation. The present findings should not be used to modify clinical management.

Several limitations must be acknowledged. First, this was a retrospective single-center study, and the residual categories were derived from tertiles of the present cohort rather than from externally validated biological or clinical thresholds. The categories and all category-related comparisons should therefore be considered exploratory and hypothesis-generating. Second, the primary residual analysis included 53 patients with detectable calcitonin, which limited precision and precluded complex modeling. Although the hypersecretory category showed nominal enrichment for lymphovascular and perineural invasion, the global three-category comparisons were not statistically significant, and the associations did not meet the conventional significance threshold after FDR adjustment. Third, the primary model did not initially account for sex or metastatic disease burden. Sensitivity analyses incorporating sex, metastatic lymph-node count, and documented M1 status and restricting the analysis to patients with pN0 disease and no documented distant metastasis supported the stability of the tumor diameter–calcitonin relationship. Nevertheless, reliable pN1a/pN1b subdivision, the size of the largest nodal metastatic deposit, and complete pTNM stage were not systematically available, and residual confounding cannot be excluded. Fourth, basal calcitonin is sex-dependent and assay-dependent. Although the sex-adjusted model showed substantial concordance with the primary model, the assay manufacturer and platform, analytical or functional sensitivity, and contemporaneous sex-specific reference intervals could not be verified reliably from the available retrospective laboratory records. Only the lower reporting limit of <2 pg/mL was consistently retrievable. Fifth, patients with serum calcitonin below the assay reporting limit were excluded from continuous category assignment. Their diagnoses were accepted from finalized institutional pathology reports, but case-level immunohistochemical panels and standardized staining-intensity data could not be reconstructed reliably. Accordingly, these patients were described as a serum-calcitonin-undetectable subgroup rather than as definitively non-secretory MTC. Finally, standardized Ki-67, mitotic count, quantitative calcitonin and CEA immunohistochemistry, granular molecular data, centralized slide review, FNAC, FNA-Ct, EU-TIRADS classification, and complete three-dimensional tumor measurements were not systematically available. Future multicenter studies should integrate these variables with standardized nodal and distant metastatic burden assessment.

Despite these limitations, the study provides a structured hypothesis-generating framework for separating the component of calcitonin variation explained by dominant tumor diameter from residual variation. Within this exploratory framework, the cohort-derived hypersecretory category showed nominal enrichment for lymphovascular and perineural invasion without meeting the FDR-adjusted significance threshold. The present findings support further investigation of tumor diameter–adjusted calcitonin secretion but do not establish a reproducible biological subtype or a clinical decision tool. Multicenter validation using standardized calcitonin assays, comprehensive nodal and distant metastatic assessment, formal pathological grading, immunohistochemical quantification, and molecular annotation is required.

## 5. Conclusions

Dominant tumor diameter explained a substantial but incomplete proportion of preoperative calcitonin variability in MTC. Exploratory, cohort-derived residual categories provided a framework for examining secretion relative to tumor diameter. The hypersecretory category showed nominal enrichment for lymphovascular and perineural invasion, but these findings did not meet the false-discovery-rate-adjusted significance threshold and should be regarded as hypothesis-generating. The tumor diameter–calcitonin relationship remained stable in sensitivity analyses accounting for sex and documented metastatic burden and in patients with pN0 disease and no documented distant metastasis. External multicenter validation using assay-specific sex adjustment, comprehensive nodal and distant metastatic assessment, standardized pathology, and molecular characterization is required before clinical application.

## Figures and Tables

**Table 1:** Baseline characteristics according to calcitonin detectability.

Variable	Overall Cohort *n* = 70	Detectable Calcitonin *n* = 53	Serum-Calcitonin-Undetectable (<2 pg/mL), *n* = 17	*p* Value
Age, years	55.5 (49.2–64.8)	57.0 (50.0–65.0)	53.0 (48.0–60.0)	0.195
Female sex	46/70 (65.7%)	33/53 (62.3%)	13/17 (76.5%)	0.383
Dominant tumor diameter, mm	10.0 (6.0–20.0)	12.0 (7.0–22.0)	4.0 (2.0–7.0)	<0.001
Total MTC tumor burden, mm	10.5 (6.0–20.0)	12.0 (7.0–22.0)	4.0 (2.0–7.0)	<0.001
Dominant tumor diameter ≤ 10 mm	36/70 (51.4%)	22/53 (41.5%)	14/17 (82.4%)	0.005
Preoperative calcitonin, pg/mL	Censored by <2 values; not summarized	124.0 (54.4–613.0)	<2 by definition	Not tested
Preoperative CEA, ng/mL	2.6 (1.3–5.1)	3.2 (1.9–7.3)	1.5 (0.5–2.3)	<0.001
Lymphovascular invasion	21/70 (30.0%)	19/53 (35.8%)	2/17 (11.8%)	0.073
Perineural invasion	9/70 (12.9%)	9/53 (17.0%)	0/17 (0.0%)	0.101
Capsular invasion	14/70 (20.0%)	13/53 (24.5%)	1/17 (5.9%)	0.162
Extrathyroidal extension	9/70 (12.9%)	9/53 (17.0%)	0/17 (0.0%)	0.101
Tumor necrosis	6/70 (8.6%)	6/53 (11.3%)	0/17 (0.0%)	0.324
Adverse pathological phenotype	24/70 (34.3%)	21/53 (39.6%)	3/17 (17.6%)	0.143
Concomitant papillary thyroid lesion	24/70 (34.3%)	14/53 (26.4%)	10/17 (58.8%)	0.020

**Table 2:** Tumor size-adjusted calcitonin secretion model and phenotype definition. (**A**) Size-adjusted calcitonin secretion model. (**B**) Residual-based secretory phenotype definition.

**(A)**				
**Model Component**			**Result**	
Primary analytic cohort			53 patients with detectable calcitonin	
Spearman correlation: dominant tumor diameter vs. calcitonin			ρ = 0.644, *p* < 0.001	
Log-linear equation			log10(calcitonin) = 0.383 + 1.712 × log10 (dominant tumor diameter)	
Slope β for log10 tumor diameter			1.712	
95% CI for slope			1.183–2.242	
Model R^2^			0.452	
Adjusted R^2^			0.442	
Overall model *p* value			<0.001	
**(B)**				
**Secretory Phenotype**	***n***	**Secretory Residual Range**	**Median Residual (IQR)**	**Median Observed/Expected Calcitonin Fold Ratio**
Hyposecretory	18	−1.998 to −0.195	−0.566 (−0.747 to −0.326)	0.27 (0.18–0.47)
Normosecretory	17	−0.122 to 0.233	0.077 (−0.043 to 0.146)	1.19 (0.91–1.40)
Hypersecretory	18	0.255 to 1.029	0.610 (0.454–0.840)	4.07 (2.85–6.91)

**Table 3:** Hypersecretory phenotype versus non-hypersecretory phenotypes for invasive pathological features.

Outcome	Hypersecretory *n* = 18	Non-Hypersecretory *n* = 35	Absolute Risk Difference	Unadjusted OR (95% CI)	Fisher Exact *p*	FDR q
Lymphovascular invasion	10/18 (55.6%)	9/35 (25.7%)	+29.8 percentage points	3.61 (1.09–11.98)	0.040	0.072
Perineural invasion	6/18 (33.3%)	3/35 (8.6%)	+24.8 percentage points	5.33 (1.15–24.79)	0.048	0.072
Adverse pathological phenotype	10/18 (55.6%)	11/35 (31.4%)	+24.1 percentage points	2.73 (0.84–8.80)	0.138	0.138

## Data Availability

The datasets generated and/or analyzed during the current study are not publicly available because they contain patient-level institutional clinical, biochemical, surgical, and pathological data and are subject to patient confidentiality and institutional data-protection regulations. De-identified data may be made available from the corresponding author upon reasonable request, subject to approval by the relevant institutional authorities and compliance with applicable ethical and data-protection requirements.

## Supplementary Materials

The following supporting information can be downloaded at: https://www.mdpi.com/article/10.3390/curroncol33090533/s1. Table S1: Nodal and distant metastatic burden across primary cohort-derived secretory categories; Table S2: Clinical and biochemical characteristics across calcitonin secretory categories; Table S3: Pathological features across calcitonin secretory categories; Table S4: Exploratory age- and sex-adjusted logistic models; Table S5: Sensitivity analysis using total MTC tumor burden instead of dominant tumor diameter; Table S6: Sex-adjusted tumor diameter–calcitonin model and category-concordance sensitivity analysis; Table S7: Sensitivity analyses accounting for nodal and distant metastatic burden.
