# Supplementary material for: Beyond Tumor Diameter: Exploratory Cohort-Derived Calcitonin Secretory Categories and Invasive Pathology in Medullary Thyroid Carcinoma
Source: Curr Oncol. 2026 Sep 2;33(9):533. doi: 10.3390/curroncol33090533 (PMC13605184; doi:10.3390/curroncol33090533)
Supplement: Supplementary file 1 [file curroncol-33-00533-s001.zip › Supplementary Table S1.pdf]

Supplementary Table S1. Nodal and distant metastatic burden across primary cohort-derived secretory categories

Variable	Hyposecretory *n* = 18	Normosecretory *n* = 17	Hypersecretory *n* = 18	p value
pN0	8/18 (44.4%)	9/17 (52.9%)	7/18 (38.9%)	0.934*
pN-positive	8/18 (44.4%)	6/17 (35.3%)	9/18 (50.0%)	
pNx	2/18 (11.1%)	2/17 (11.8%)	2/18 (11.1%)	
Metastatic lymph nodes, median (IQR)	0 (0–2)	0 (0–1)	2 (0–9.75)	0.269
Total lymph nodes examined, median (IQR)	5 (3.25–18.25)	5 (2–13)	26.5 (10.75–43)	0.008
Documented M1 disease	4/18 (22.2%)	0/17 (0.0%)	4/18 (22.2%)	0.108

*The p value represents the global comparison of pN0, pN-positive, and pNx distributions. pN0 was defined as at least one examined lymph node with no metastasis; pN-positive as at least one metastatic lymph node; and pNx as no pathological lymph-node assessment. The number of lymph nodes examined reflects the extent of clinically indicated neck dissection and should not be interpreted as an intrinsic measure of tumor burden. IQR, interquartile range.
