# Supplementary material for: Beyond Tumor Diameter: Exploratory Cohort-Derived Calcitonin Secretory Categories and Invasive Pathology in Medullary Thyroid Carcinoma
Source: Curr Oncol. 2026 Sep 2;33(9):533. doi: 10.3390/curroncol33090533 (PMC13605184; doi:10.3390/curroncol33090533)
Supplement: Supplementary file 1 [file curroncol-33-00533-s001.zip › Supplementary Table S2.pdf]

Supplementary Table S2. Clinical and biochemical characteristics across calcitonin secretory categories

Variable	Hyposecretory *n* = 18	Normosecretory *n* = 17	Hypersecretory *n* = 18	p value
Age, years	60.0 (52.2–64.5)	57.0 (53.0–69.0)	52.0 (49.0–60.2)	0.282
Female sex	13/18 (72.2%)	13/17 (76.5%)	7/18 (38.9%)	0.049
Calcitonin, pg/mL	49.1 (18.9–95.9)	123.0 (82.5–226.0)	2000.0 (398.5–4763.0)	<0.001
CEA, ng/mL	2.6 (1.4–4.0)	2.6 (1.9–4.7)	5.8 (2.9–26.1)	0.041
Dominant tumor diameter, mm	11.5 (8.2–19.8)	9.0 (6.0–14.0)	17.5 (9.2–30.0)	0.127
Total MTC tumor burden, mm	11.5 (8.2–19.8)	10.0 (6.0–15.0)	17.5 (9.2–37.0)	0.148
Calcitonin secretory density, pg/mL/mm	4.1 (2.3–5.4)	13.8 (10.8–16.1)	86.1 (44.8–153.4)	<0.001
CEA/calcitonin ratio	0.073 (0.020–0.141)	0.028 (0.018–0.043)	0.007 (0.003–0.021)	<0.001
Secretory residual, log10 units	−0.566 (−0.747 to −0.326)	0.077 (−0.043 to 0.146)	0.610 (0.454–0.840)	<0.001
Observed/expected calcitonin fold ratio	0.27 (0.18–0.47)	1.19 (0.91–1.40)	4.07 (2.85–6.91)	<0.001

Values are median (IQR) or n/N (%). p values represent global three-group comparisons.
CEA, carcinoembryonic antigen; MTC, medullary thyroid carcinoma.
