# Supplementary material for: Beyond Tumor Diameter: Exploratory Cohort-Derived Calcitonin Secretory Categories and Invasive Pathology in Medullary Thyroid Carcinoma
Source: Curr Oncol. 2026 Sep 2;33(9):533. doi: 10.3390/curroncol33090533 (PMC13605184; doi:10.3390/curroncol33090533)
Supplement: Supplementary file 1 [file curroncol-33-00533-s001.zip › Supplementary Table S3.pdf]

Supplementary Table S3. Pathological features across calcitonin secretory categories

Pathological feature	Hyposecretory *n* = 18	Normosecretory *n* = 17	Hypersecretory *n* = 18	p value
Lymphovascular invasion	5/18 (27.8%)	4/17 (23.5%)	10/18 (55.6%)	0.135
Perineural invasion	2/18 (11.1%)	1/17 (5.9%)	6/18 (33.3%)	0.117
Capsular invasion	5/18 (27.8%)	3/17 (17.6%)	5/18 (27.8%)	0.783
Tumor necrosis	4/18 (22.2%)	1/17 (5.9%)	1/18 (5.6%)	0.339
Extrathyroidal extension	5/18 (27.8%)	1/17 (5.9%)	3/18 (16.7%)	0.259
Adverse pathological phenotype	6/18 (33.3%)	5/17 (29.4%)	10/18 (55.6%)	0.264

Values are n/N (%). p values represent global three-group comparisons. Adverse pathological phenotype was defined as the presence of at least one of the following: lymphovascular invasion, perineural invasion, capsular invasion, tumor necrosis, or extrathyroidal extension.
