# Supplementary material for: Beyond Tumor Diameter: Exploratory Cohort-Derived Calcitonin Secretory Categories and Invasive Pathology in Medullary Thyroid Carcinoma
Source: Curr Oncol. 2026 Sep 2;33(9):533. doi: 10.3390/curroncol33090533 (PMC13605184; doi:10.3390/curroncol33090533)
Supplement: Supplementary file 1 [file curroncol-33-00533-s001.zip › Supplementary Table S4.pdf]

Supplementary Table S4. Exploratory age- and sex-adjusted logistic models

Outcome	Hypersecretory *n* = 18	Non-hypersecretory *n* = 35	Age/sex-adjusted OR (95% CI)	Adjusted p value
Lymphovascular invasion	10/18 (55.6%)	9/35 (25.7%)	3.84 (1.01–14.62)	0.049
Perineural invasion	6/18 (33.3%)	3/35 (8.6%)	7.00 (1.17–41.83)	0.033
Adverse pathological phenotype	10/18 (55.6%)	11/35 (31.4%)	3.12 (0.84–11.57)	0.088

These models are exploratory and should be interpreted cautiously because of limited event numbers. The primary inferential table is Table 3, which reports unadjusted odds ratios, Fisher exact p values, and FDR-adjusted q values.
CI, confidence interval; OR, odds ratio.
