# Supplementary material for: Beyond Tumor Diameter: Exploratory Cohort-Derived Calcitonin Secretory Categories and Invasive Pathology in Medullary Thyroid Carcinoma
Source: Curr Oncol. 2026 Sep 2;33(9):533. doi: 10.3390/curroncol33090533 (PMC13605184; doi:10.3390/curroncol33090533)
Supplement: Supplementary file 1 [file curroncol-33-00533-s001.zip › Supplementary Table S5.pdf]

Supplementary Table S5. Sensitivity analysis using total MTC tumor burden instead of dominant tumor diameter

Analysis	Dominant tumor diameter model	Total tumor burden model
Spearman correlation with calcitonin	ρ=0.644, p<0.001	ρ=0.657, p<0.001
Log-linear slope β	1.712	1.699
95% CI for slope	1.183–2.242	1.193–2.205
Model R^2	0.452	0.471
Adjusted R^2	0.442	0.461
Overall model p value	<0.001	<0.001
Concordance of category classification	Reference	51/53 (96.2%)
Cohen's κ for category classification	Reference	0.943
Hypersecretory classification stability	Reference	18/18 unchanged

Total MTC tumor burden was calculated as the sum of reported MTC focus diameters in multifocal cases.
CI, confidence interval; MTC, medullary thyroid carcinoma.
