# Supplementary material for: Beyond Tumor Diameter: Exploratory Cohort-Derived Calcitonin Secretory Categories and Invasive Pathology in Medullary Thyroid Carcinoma
Source: Curr Oncol. 2026 Sep 2;33(9):533. doi: 10.3390/curroncol33090533 (PMC13605184; doi:10.3390/curroncol33090533)
Supplement: Supplementary file 1 [file curroncol-33-00533-s001.zip › Supplementary Table S6.pdf]

Supplementary Table S6. Sex-adjusted tumor diameter–calcitonin model and category-concordance sensitivity analysis

Panel A

Model component	Estimate	95% CI	p value
Intercept	0.395	−0.208 to 0.999	0.194
log10 dominant tumor diameter	1.595	1.057 to 2.134	<0.001
Male sex	0.312	−0.057 to 0.681	0.096
Model R^2	0.482		
Adjusted R^2	0.461		

Panel B

Concordance metric	Result
Overall concordance	45/53 (84.9%)
Cohen’s kappa	0.774
Original hypersecretory cases remaining hypersecretory	16/18 (88.9%)

The sex-adjusted residual categories were derived using tertiles of residuals from a model including log10 dominant tumor diameter and male sex. The analysis was exploratory.
