# Supplementary material for: Beyond Tumor Diameter: Exploratory Cohort-Derived Calcitonin Secretory Categories and Invasive Pathology in Medullary Thyroid Carcinoma
Source: Curr Oncol. 2026 Sep 2;33(9):533. doi: 10.3390/curroncol33090533 (PMC13605184; doi:10.3390/curroncol33090533)
Supplement: Supplementary file 1 [file curroncol-33-00533-s001.zip › Supplementary Table S7.pdf]

Supplementary Table S7. Sensitivity analyses accounting for nodal and distant metastatic burden

Panel A: Metastatic-burden-adjusted model, *n* = 47

Model component	Estimate	95% CI	p value
Intercept	0.470	−0.273 to 1.212	0.209
log10 dominant tumor diameter	1.447	0.734 to 2.161	<0.001
Male sex	0.333	−0.071 to 0.736	0.104
log(1 + metastatic lymph-node count)	0.172	−0.048 to 0.393	0.122
Documented M1 status	−0.332	−0.897 to 0.232	0.241
Model R^2	0.483		
Adjusted R^2	0.434		

Panel B: Concordance

Concordance metric	Result
Overall concordance	40/47 (85.1%)
Cohen's kappa	0.776

Panel C: pN0 and no documented distant metastasis, *n* = 23

Model component	Estimate	95% CI	p value
Intercept	0.120	−0.699 to 0.940	0.763
log10 dominant tumor diameter	1.912	1.160 to 2.664	<0.001
Model R^2	0.571		
Adjusted R^2	0.550		

Only two lymphovascular invasion events and no perineural invasion events occurred in the restricted pN0 subgroup without documented distant metastasis; therefore, no secretory-category versus pathology analysis was performed.
